# Sensors in Multiple Sclerosis

**DOI:** 10.1007/s11910-025-01448-0

**Published:** 2025-10-29

**Authors:** Angeliki G. Filippatou, Ellen M. Mowry

**Affiliations:** Division of Neuroimmunology and Neurological Infections, Department of Neurology, Johns Hopkins University School of Medicine, 600 N. Wolfe St, Baltimore, MD 21287 USA

**Keywords:** Biosensors, Digital biomarkers, Digital tools, Wearables, Multiple sclerosis

## Abstract

**Purpose of Review:**

Biosensors and digital tools may enhance monitoring of people with multiple sclerosis (MS) and support timely, data-driven clinical decisions. We review current and emerging applications of biosensors to monitor function in MS.

**Recent Rindings:**

Biosensors track diverse physiological and kinetic metrics, allowing assessment of function across several key domains in MS, including physical activity, circadian rhythmicity, gait, balance, fine motor function, and bladder control. A consistent cross-study finding is that novel technologies reliably capture subtle abnormalities that are often missed by traditional assessment methods.

**Summary:**

Digital health technologies hold significant promise for transforming MS care by enabling precise, continuous monitoring of functional status and disease progression. They may facilitate personalized management, allowing clinicians to tailor interventions based on each person’s unique disease trajectory. Further studies are essential to validate the predictive value and responsiveness of these tools and ensure their effective integration into clinical practice and trials.

## Introduction

Multiple sclerosis (MS) is a complex immune-mediated disease of the central nervous system. In most patients, MS has an initial relapsing course caused by autoimmune-induced demyelination, often followed by a progressive course characterized by gradual disability accumulation, caused by neurodegeneration [1]. A subset of people with MS (PwMS) have a primary progressive course, wherein disability accumulates gradually from disease onset. Therapeutic development in progressive MS has been hampered by a lack of reliable tools to monitor disease progression. The Expanded Disability Status Scale (EDSS) is widely used and is considered the gold standard for phase 3 clinical trials in progressive MS by regulatory agencies. However, it is a semi-quantitative, non-linear scale with limited reliability and sensitivity to change over short timeframes [2–4]. Moreover, clinical assessments typically occur only once every three to six months, providing a limited snapshot of an individual’s functioning.

Recent advancements in digital health technologies may transform the monitoring and management of PwMS by enabling more precise, continuous, and quantitative assessments of functional status and disease progression, paving the way for individualized care strategies [5, 6]. Several studies have investigated biosensors as tools to provide objective and sensitive measures of disability in MS. Digital biosensors convert physiological information into digital data, which can be subsequently analyzed [7]. Various types of digital biosensors can be applied to obtain data on multiple domains of function in MS. Sensors are often embedded into wearable devices that collect data via passive monitoring (while an individual is wearing the device during their habitual daily routines) or during active tasks (while an individual is performing a specific task designed to measure key functions).

In this narrative review, we summarize current and emerging applications of biosensors to monitor function in MS. We provide an overview of commonly used sensor technologies and their key applications in each functional domain, including overall physical activity (PA) and circadian rhythmicity (CR), gait, balance, fine motor function, and bladder function.

## Sensor Technologies Overview

Commonly used biosensor technologies are summarized in Table 1. Accelerometry has emerged as a prominent modality in biosensor research. Accelerometers detect body motion-induced accelerations; this is typically accomplished through a small internal mass that moves in response to acceleration. These devices can detect one-, two-, or three-dimensional linear acceleration. Original studies in MS applied uniaxial accelerometers or pedometers to obtain step counts as a metric to track daily mobility [8]. Accelerometers remain among the most widely used sensor types, with recent studies employing triaxial accelerometers to track the duration, frequency and intensity of PA [7, 9]. Accelerometers can be augmented by gyroscopes, which measure one-, two-, or three-dimensional angular velocities to capture the rotational motion of the body segment to which they are attached. The combination of multi-dimensional accelerometers, gyroscopes and often magnetometers compose the inertial measurement units (IMUs) [10]. The inclusion of magnetometers improves orientation tracking and particularly heading, which refers to the angle of the sensor with respect to the horizontal direction of the magnetic north. These sensors are often embedded into wearable devices such as motion trackers/ smartwatches or smartphones and can be used to monitor PA, gait, balance, and dexterity, among others. Commercial activity trackers that often include photoplethysmography (PPG) sensors (e.g. Fitbit, Garmin) have also been integrated into research protocols, as they offer a promising avenue for scalable and cost-effective digital biomarkers that can be deployed in large cohorts [11]. Multi-sensors which integrate accelerometers, gyroscopes and additional modalities such as surface electromyogram (sEMG) may provide a more comprehensive evaluation of muscle activity and movement [12]. In addition to these tools, a diverse array of specialized sensors has been applied to capture more detailed or domain-specific functional impairments in MS; these will be further discussed herein.

**Table 1:** Common wearable sensor technologies

Type of wearable sensor	Description
Accelerometer	Detection of one-, two-, or three-dimensional linear acceleration
Gyroscope	Detection of one-, two-, or three-dimensional angular velocities
Magnetometer	Measurement of magnetic fields, often used to enhance orientation tracking when combined with other sensors
Inertial measurement unit (IMU)	Combination of multi-dimensional accelerometer, gyroscope and magnetometer
Insoles with pressure sensors	Measurement of pressure distribution and changes between the foot and ground
Photoplethysmography (PPG)	Optical measurement of blood volume changes, often used to infer heart rate and blood oxygen levels

## Monitoring of Function across Key Domains

### Physical Activity, Sleep and Circadian Rhythmicity

Wearable motion sensors, particularly accelerometers, have allowed the remote, objective monitoring of multiple dimensions of movement, including step counts, intensity of PA, and measures of sleep and CR. Initial studies in MS focused on pedometers and accelerometers to obtain daily activity counts or step counts as surrogate markers for PA [8]. Wearable devices are commonly placed on the waist, wrist, upper leg or ankle and can be used for monitoring in both real-world and laboratory environments [13]. Most real-world studies in MS typically equip participants with a single wearable device, whereas laboratory-based studies often utilize multiple devices placed on various parts of the body [13]. Accelerometers have demonstrated good to excellent reliability across a range of devices, including both research-grade and commercial models [7, 14]. Step count accuracy differs by body-worn sensor location; it is typically highest at the ankle, followed by waist-worn and wrist-worn devices, both in MS and healthy control (HC) populations [15–18]. However, wrist-worn devices offer several advantages for studies focused on outcomes beyond step count, including higher participant adherence and the ability to capture activity in non-ambulatory individuals.

PwMS have been found to have lower average levels of PA than controls [19]. Among PwMS, lower PA has been associated with higher disability scores and worse performance across a range of clinical outcomes, including walking speed and endurance (e.g. timed 25-foot walk [T25FW], 2-minute walk test [2MWT], 6-minute walk test [6MWT]), and balance (e.g. timed-up-and-go [TUG]), as well as with patient-reported outcomes (PROs; e.g. fatigue, depression, self-efficacy) [13, 19]. People with progressive MS (PwPMS) take fewer average daily steps compared to people with relapsing MS (PwRMS) [14]. In a longitudinal study of 306 PwMS, those who experienced disability progression over 24 months had a lower number of daily steps [20]. Similarly, in a longitudinal study of 95 PwMS, decreasing total step count over one year was associated with worse clinic-based assessments (T25FW, TUG) and PROs [21]. Limitations of these wearable motion sensors include potentially reduced accuracy in step recording for participants with greater levels of disability and slower gait [9, 18]. Moreover, activity and step counts may differ between devices, rendering cross-device comparisons challenging [22, 23].

In addition to global activity metrics, accelerometers can be used to measure intensity of free-living PA and sedentary time. A recent meta-analysis of 21 studies leveraging accelerometers in MS found that PwMS are more sedentary and engage in less light PA or moderate-to-vigorous PA than HC [24]. These differences are accentuated with advanced disability, compared to both HC and less disabled PwMS [25]. A study in pediatric MS found that children with MS spent significantly less time in moderate-to-vigorous PA compared to HC [26]. A key limitation of the existing literature on PA in MS is the significant methodological heterogeneity, including use of different analytic methods to derive PA metrics from accelerometry data, which complicates cross-study comparisons [27].

Accelerometry can be used to track not only overall PA, but also more nuanced behavioral patterns, including measures of activity fragmentation, CR and sleep. Activity fragmentation refers to the degree in which active periods are interrupted by transitions between activity and inactivity; higher fragmentation often reflects reduced endurance. Measures of activity fragmentation differ between PwMS and HC, reflecting more fragmented activity in PwMS, and are associated with disability [28]. PwPMS exhibit more fragmented activity than PwRMS with higher active-to-sedentary transition probability and weaker CR [29]. With regards to sleep, PwMS have been shown to have lower sleep efficiency and sleep regularity compared to controls, and correlations have been found between cognitive function and measures of sleep fragmentation and quality [30, 31]. Beyond accelerometer-derived activity measures, commercial wearable devices offer additional sensors such as PPG sensors for monitoring of autonomic functions and sleep tracking algorithms for monitoring of sleep stages, however they have not been validated for use in the clinic. A small cross-sectional study examined autonomic data in PwMS with moderate disability and found that their median heart rate was higher compared to controls and positively correlated with EDSS and T25FW [32]. Commercial wearable sleep algorithms have not been specifically validated in PwMS and, even in healthy individuals, they often misclassify wake periods after sleep onset as sleep and have mixed performance in detecting sleep stages [33–35]. The limitations of commercial devices are likely to be exaggerated in PwMS, where periods of restful wakefulness (possibly due to reduced mobility or other factors) may be misclassified as sleep or autonomic dysfunction may distort physiological signals.

While the findings from studies described above provide valuable insights into PA patterns in MS, they are primarily based on aggregated data summaries – typically averaging PA intensity or duration over 24-hour periods. This approach does not account for diurnal activity fluctuations. However, accelerometry captures data at a much finer temporal resolution (sub-minute level), and many analytical methods fail to fully leverage the richness of actigraphy data [36]. In fact, a prior study from our center reported significant diurnal variability in accelerometry-measured PA across 24-hours and found unique relationships between PA at specific times of day and global disability by using time-of-day specific linear models and function-on-scalar regression [37]. Moreover, in a recent paper reporting the baseline data from our longitudinal observational study HEAL-MS (Home-based Evaluation of Actigraphy to predict Longitudinal function in MS), our group found that the lower PA in PwPMS vs. PwRMS was mainly driven by activity differences during specific times of the day [29]. Our group also applied a novel integrative dimension reduction technique, the Joint and Individual Variation Explained (JIVE) model, to assess the co-dependencies between PA and CR patterns and their joint and individual associations with MS subtype. The analysis revealed overlap between variation in PA and CR; PwPMS had higher first joint component, which was associated with both total volume of PA and measures of strength of CR [29]. These findings highlight the interdependence between these domains and their combined relevance to MS subtypes.

In a follow-up longitudinal study of the HEAL-MS cohort, our group found that changes in activity timing in the 24-hour cycle were significantly associated with brain atrophy on standardized MRI scans. Within-person increases in night-time activity, and within-person decreases of morning activity, were associated with faster rates of whole brain and gray matter volume loss over a mean follow-up of 1.17 years, suggesting that these technologies may predict worse outcomes at the individual level [38]. In a study utilizing a multi-sensor (tri-axial accelerometer and sensors that measure heat flux, galvanic skin response and skin temperature) to estimate daily step count, metabolic equivalents, PA duration, and active energy expenditure, decrease in the composite PA score was associated with faster rates of brain atrophy over 30-month follow-up [39]. These findings provide evidence for a potential link between wearable-derived digital biomarkers and ongoing neurodegeneration, as captured by brain atrophy.

### Gait

A promising application of biosensors in MS is for assessment of gait. Gait dysfunction represents a critical aspect of MS-related disability, and gait performance provides insight into the integrity of multiple pathways including pyramidal, cerebellar and sensory. Traditional assessments of gait, such as T25FW or the ambulation assessment as part of the EDSS, rely on coarse measures such as short-distance walking speed or total distance walked, which do not adequately capture the complex and multidimensional nature of gait performance. Several movement analysis technologies have been utilized, including both non-wearable and wearable sensors, with the aim of characterizing and quantifying gait performance using measures of gait speed, stability and smoothness.

Non-wearable sensors to capture gait include optical motion capture technologies, force platforms and instrumented walkway mats. Optical motion capture systems are either marker-based or marker-less and measure kinematics of gait in three dimensions [40]. Marker-based systems are based on optoelectronic stereophotogrammetry and use reflective markers placed on anatomical landmarks, such as joints, to capture motion of body parts. The assessments need to be performed in dedicated spaces such as gait laboratories. Marker-based optical motion capture systems have been utilized in small studies in PwMS and have shown reduced gait speed, stride length, prolonged double support time and associations with disease severity [40]. In a small study of prolonged-release fampridine, a medication used for gait fatigue in MS, gait pattern changes were found at the single-subject level based on 3D gait analysis during treadmill walking and correlated with improvements in T25FW and 6MWT [41]. Marker-less systems use depth sensors or video cameras to capture movement; machine learning algorithms reconstruct 3D motion data [10, 42]. Ambient measurement systems, a type of marker-less technology, have been applied in the homes of PwMS to estimate average and peak walking speed and have shown correlations with clinic-based assessments of gait, however their utility remains limited to motion within the confines of the home environment [43]. Force platforms, which are equipped with sensors to measure ground reaction forces, represent another key technology to study changes in gait initiation, postural stability, and balance. Their use, however, is limited due to cost and need for dedicated laboratory environment [40]. Instrumented walkway mats (e.g. GAITRite) are portable mats with sensors to identify foot contacts and measure spatiotemporal measures of gait such as walking speed, step and stride lengths, and base of support. Measures of gait impairment in PwMS have shown associations with T25FW, EDSS, cerebellar functional system score, free-living accelerometry-derived activity counts, and PROs; subtle gait abnormalities have been found even in individuals with early MS [40, 44]. A key limitation of these technologies is the ability to capture only a few steps at a time.

Wearable sensors offer possible advantages over non-wearable systems for gait analysis, including portability and ability for continuous monitoring in real-world environments. Wearable technologies for gait evaluation include accelerometers, IMUs, instrumented insoles with pressure sensors, as well as multi-sensors. Common locations for placement of accelerometers and IMUs include wrist, trunk, waist, thigh, shank, ankle; skin-mounted wireless adhesive inertial sensors have also been developed to improve patient comfort [13, 45, 46]. Studies have utilized a single sensor or multiple sensors in different body parts to capture and characterize gait through active or passive monitoring. To extend monitoring capabilities to PwMS who require assistive devices for walking, a sensorized tip that can be attached to a crutch or cane has been proposed [47]. Wearable sensor data suggest changes in gait stability and regularity in PwMS that correlate with disability levels, but such changes may also be seen in individuals with early MS without disability [48, 49]. In a study comparing in-lab and community ambulation using a tri-axial lower back accelerometer, gait speed during most of community ambulation was lower than in-lab values, and PwMS took fewer steps and had slower gait speed and larger stride-to-stride variability in both environments compared to controls [50]. Metrics derived from real-world accelerometry (e.g. maximum step rate, habitual step rate, peak cadence) have been proposed as potential clinically relevant metrics and show associations with in-clinic assessments such as 6MWT and measures of disability [51, 52]. Moreover, wearable sensors have been utilized for instrumented in-clinic assessments of gait, including instrumented T25FW, 2MWT and 6MWT [53–56]. Several studies have quantified gait parameters during the 6MWT in PwMS and demonstrated worsening in gait characteristics throughout the walk, including decrease in speed, cadence, step length, stability and regularity [53, 57, 58]. In a longitudinal observational study, PwMS who had slower gait speed and a flattened gait speed trajectory curve during baseline 6MWT had a higher risk of worsening across clinical metrics and PROs during 2-year follow-up [59]. In a separate longitudinal study, changes in gait instability (stride regularity and medio-lateral gait symmetry) were associated with changes in EDSS and decrease in subjective perception of stability over a 2-year period [60]. Improvements in spatiotemporal gait parameters have been observed following rehabilitation programs [61, 62].

Instrumented insoles and multi-sensors have also emerged as tools to evaluate gait. Instrumented insoles have integrated pressure sensors to measure pressure changes between the foot and ground, utilizing technology similar to force platforms [40]. Instrumented insoles may also contain motion sensors such as accelerometers and gyroscopes themselves or may be utilized as part of multi-sensor systems to evaluate gait [63–65]. In MS, these sensors have been utilized in small studies, and their accuracy has been validated against instrumented walkways and stereophotogrammetry [63, 64]. Multi-sensors have also been proposed to enrich data collected from wearables. A study of 25 PwMS utilized the Cardiac and Activity Monitor (CAM) device, which incorporates IMU, PPG and additional sensors to measure skin impedance, body temperature, and environmental factors (e.g. light exposure, air pressure) [66]. Several CAM biosensor-derived gait and balance metrics correlated with EDSS and MS functional composite (MSFC) scores.

In recent years, studies have leveraged tri-axial accelerometers and gyroscopes embedded in smartphones to develop smartphone applications for objective gait assessments [67]. Proposed smartphone-based gait assessments include active tests such as the 2MWT and the 5 U-Turn Test (5UTT; measuring angular velocity during 5 U-turns while walking) [68–71]. One study has applied smartphone-based gait analysis to evaluate fatigability (decline in gait performance) during a 6MWT in PwMS [72]. Several gait tasks have been incorporated into the Floodlight application, which has been proposed as a tool to allow remote monitoring of function across multiple domains in PwMS [73, 74]. Gait assessments within this app include both passive monitoring of gait and active tests (2MWT, 5UTT) [75]. These active gait assessments have demonstrated adequate test-retest reliability and have shown correlations with T25FW and EDSS; additionally, there were correlations between gait measures obtained through active and passive monitoring [75]. Smartphone-based digital health applications may offer scalable and accessible solutions to remotely monitor functional performance in MS, but further research is needed to validate their clinical utility.

### Balance and Falls

Biosensors have emerged as valuable tools to measure balance and postural control. Traditionally, evaluations relied on static and dynamic posturography using force plates. However, wearable sensors have gained attention as more accessible and versatile alternatives [76]. Postural sway metrics from wearable accelerometers and IMUs have been validated against force plates [77, 78] and have been utilized for instrumented versions of balance tasks such as tandem gait, Romberg, single leg stance, stair ascent and TUG, showing balance impairments in PwMS compared to controls [79–84]. Turning speed during instrumented TUG has shown associations with gray matter brain volumes, while postural sway metrics during standing tasks were correlated with diffusion MRI-derived metrics of structural integrity of the cerebellar peduncles [85, 86]. Smartphone applications have also been utilized to evaluate balance in MS [87]. These include the Mon4t app, which features TUG and tandem gait tasks, the Floodlight app, which includes a Static Balance Test (measuring sway while standing unsupported for 30 s), and the elevateMS app, which incorporates a walk and balance test. Metrics of posture, stability and balance collected from these applications have demonstrated correlations with traditional metrics of disability in cross-sectional studies of PwMS [75, 88, 89].

Biosensor-derived measures of balance have been proposed as potential predictors of fall risk in MS. Quantitative evaluation of instrumented gait and balance tasks, as well as passive gait monitoring in PwMS, have shown that fallers tend to have worse metrics, including increased sway area and jerk, fewer daily steps, altered thigh acceleration during sit-to-stand transitions, and altered spatiotemporal measures of gait [55, 90–93]. In examining how falls occur, a study evaluating movement behaviors prior to falls found that more pauses-while-walking and more complex movement trajectories were associated with increased fall risk [94]. Smartphone-based fall risk screening apps have also been developed [95]. Thus, digital tools have the potential to enhance fall prevention by enabling fall risk stratification and personalized fall risk screening, with the goal to reduce falls and related morbidity.

### Upper Limb and Fine Motor Function

Impaired fine motor function in common in PwMS. Biosensors have been utilized to “quantify” the neurologic exam and capture precise data on movement patterns, strength, coordination, and tremor. An instrumented version of the finger-to-nose test using IMUs revealed asynchrony of the inter-hand interval in PwMS with high disability [96]. During instrumented hand- and foot-tapping using IMUs, inter-tap interval differed between PwMS and controls; foot tapping was further prolonged in PwPMS compared to PwRMS [97]. Using an alternative technology involving a finger and foot tapping platform, PwPMS demonstrated fewer finger and foot taps, which correlated with conventional disability measures such as the 9-Hole Peg Test (9HPT), T25FW and EDSS [98]. Methodologies have also been proposed to quantify upper limb tremor in MS based on wrist accelerometers [99].

Multi-sensor systems may offer a more thorough assessment of muscle activity and movement. The wearable multi-sensor MYO-band combines an accelerometer, gyroscope and sEMG and can be used to extract waveform‐based textural features during finger and foot taps to quantify limb performance; the derived metrics differ between relapsing and secondary progressive MS and are associated with EDSS [12]. In a small longitudinal study, while most PwMS had no change in EDSS during brief follow‐up, several had evidence of progression by the multi-sensor metrics [100].

Smartphone or tablet-based applications have also emerged as tools to quantify upper limb function in MS. These include digital adaptations of traditional assessments such as the Manual Dexterity Test; a tablet-based tool that simulates the 9HPT - this test is a component of the Multiple Sclerosis Performance Test (MSPT), which itself is designed to mirror the MSFC [101]. Similarly, the MSCopilot was also developed based on four assessments inspired by the revised MSFC-4, including a dexterity task of shape drawing [102]. Additional app-based tasks to evaluate fine motor skills and coordination include adaptations of finger-tapping and finger-to-nose tests, as well as novel activities such as pinching, balloon popping or dexterity challenges using a gaming interface [88, 103, 104]. Metrics of performance in these app-based tasks have demonstrated correlations with metrics of disability in MS, such as the MSFC (mainly the 9HPT component), EDSS, and PROs [88, 104, 105]. The previously mentioned Floodlight app’s [75] spatiotemporal metrics in the shape-drawing (e.g. trace accuracy, velocity variability) and pinching (e.g. double touch synchrony) tasks were correlated with clinical outcomes and brain volumetrics [106–108]. Smartphone-based applications of fine motor performance have also identified subtle differences between people with radiologically isolated syndrome and HC, highlighting the potential of digital biomarkers to identify early functional impairments in MS [109]. Limitations of smartphone-based tests include long-term practice effects, which may delay the ability to identify disease-related changes in longitudinal studies or lead to underestimation of disease progression [110].

Another noteworthy concept is the evaluation of keystroke dynamics (KD) in MS. Typing is a common daily task which requires multiple skills, including dexterity, hand-eye coordination and cognitive functions such as information processing and attention [111]. Quantitative analysis of press and release keyboard interactions allows the identification of “typing signatures,” which is hypothesized to reflect motor and non-motor functioning in MS. KD are evaluated via a smartphone app that replaces the default keyboard. KD differ between PwMS and controls, and keystroke latencies are associated with clinical outcome measures including EDSS, 9HPT and cognitive tests [111]. KD are also associated with brain volumetrics cross-sectionally, including whole brain, thalamic and lesion volumes [112]. Changes in KD have been observed in parallel with changes in disease activity, fatigue, and clinical disability in MS and KD have been longitudinally associated with MS clinical outcomes, including the 9HPT [113, 114]. In a longitudinal study of 59 PwMS, lower mean keys per second during the initial month of assessment were associated with a higher risk of disability progression within the next year [115]. Smartphone-based strategies including KD may have value for monitoring of MS.

### Bladder

Bladder dysfunction is a prevalent symptom in PwMS and may significantly compromise their quality of life. The integration of novel technologies into bladder management strategies offers promising avenues to enhance both diagnosis and treatment. Wearable and implantable devices utilize intravesical pressure measurements or non-invasive ultrasound-based technology to continuously monitor bladder fullness and alert patients about the need to void [116]. Devices with biosensors may also be used for therapeutic interventions, including assisted pelvic floor muscle training through biofeedback mechanisms and remote neuromodulation [116]. Remote neuromodulation involves subcutaneous implantable devices that stimulate tibial nerve fibers; this stimulation is hypothesized to inhibit the micturition reflex by modulating neural pathways in the spinal cord and the pelvic/pudendal nerves to help manage symptoms of overactive bladder [117].

## Limitations of Existing Research

Several current studies employing biosensors in MS face limitations. A key limitation is the reliance on small cohorts and cross-sectional or case-control designs. Such approaches hinder the ability to capture longitudinal trajectories of change in sensor-derived digital biomarkers, thereby providing limited insight into their potential to reflect disease progression or treatment responses. Moreover, use of small cohorts may limit generalizability of the results to broader MS populations. Another limitation is the heterogeneity between studies and lack of standardized protocols regarding both data acquisition protocols (e.g. frequency, duration, context of sensor-based assessments) and analytical methodology (e.g. data processing pipelines, feature extraction techniques, outcome definitions). Moreover, many commercial devices rely on proprietary, “black box” algorithms, that further limit reproducibility across platforms. The applicability of commercial tools in MS populations has its own set of unique challenges. Most algorithms have been trained on data from healthy populations and their accuracy may be lower in PwMS due to disease-specific factors; for example, usage of wearables on limbs affected by weakness or tremor may lead to inaccurate measurements due to reduced voluntary movements or involuntary motion artifacts respectively. Additionally, gait algorithms may be influenced by factors such as slower gait speed, gait asymmetry or use of assistive devices in PwMS; prior studies have shown reduced accuracy in step recording for participants with higher disability [9, 18]. Long-term data reliability from digital tools may also be compromised by variable adherence: PwMS frequently experience disease fluctuations, progression of physical disability or non-motor symptoms such as depression, fatigue and cognitive impairment, all of which may reduce their motivation and ability to consistently engage with the technology. Due to these limitations, despite a growing interest in digital tools, there remains a gap in their adoption and wide-scale implementation in both clinical care and research.

An essential next step is establishing a clear pathway for regulatory approval of digital tools in MS. This involves selecting key digital outcomes that can be rigorously validated (through assessments of accuracy, reliability, sensitivity, minimal clinically important differences and responsiveness), identifying appropriate measurement approaches for those outcomes (e.g. sensor types, timing and frequency of assessments) and establishing scalable and secure platforms for data analysis and integration [118, 119]. Given the variety of tools available as outlined therein, additional consideration is required when evaluating active and passive monitoring approaches. Passive monitoring collects data unobtrusively in real-world environments and offers higher ecological validity, while active monitoring involves structured instrumented tasks in controlled settings that may offer easier implementation. Ultimately, a combination of both approaches may offer a more comprehensive assessment of functional status in PwMS [120]. In other fields of neurology such as movement disorders, digital measures derived from wearables/mobile technologies are already being used as exploratory outcome measures in clinical trials [121, 122]. In Duchenne muscular dystrophy, the wearable-derived stride velocity 95th centile has received qualification from the European Medicines Agency (EMA) as a primary endpoint for clinical trials [123]. Similar advances have not yet been realized in MS research but there are ongoing efforts to harmonize and validate digital outcomes across multiple disease states, including MS (e.g. Mobilise-D EU initiative) [124, 125]. Guidelines for the use of biosensor technologies in clinical trials have been developed by regulatory bodies including the Food and Drug Administration (FDA) and EMA and can serve as a foundational framework for the development of disease-specific digital tools for MS [126, 127].

## Conclusions

The multisymptomatic nature of MS and the complex and often heterogeneous disease course present a wide array of opportunities for the use of digital tools and sensors. In this manuscript, we have highlighted the capability of biosensors to capture a wide range of physiological and kinetic metrics, enabling monitoring of functional impairments across several key domains in MS. Digital tools and sensors have gained significant traction, as reflected by the large number of studies applying these tools in both real-world environments and controlled clinical or laboratory settings, alongside a surge in the development and use of smartphone/ smartwatch applications [13]. Such technologies have the potential to detect subtle abnormalities that may go unnoticed with traditional assessments and can complement conventional clinical evaluations.

Digital tools and sensors may serve dual roles. First, they enable real-time, frequent assessments that may identify disease worsening earlier, improve risk stratification in PwMS and support personalized management, allowing clinicians to tailor interventions to individual disease trajectories. Second, these technologies offer a more precise means to track treatment response to interventions. This is valuable not only in routine clinical practice, but also in clinical trials, where the use of more precise outcome measures may accelerate the development of effective therapeutic strategies. To advance the field, further research is essential, particularly longitudinal studies aimed at validating the predictive value and responsiveness of digital tools, as well as efforts to standardize protocols and analytical methodologies. Despite ongoing challenges, these tools hold great potential to enable individualized, data-driven care in MS.

## Key References

- Woelfle T, Bourguignon L, Lorscheider J, Kappos L, Naegelin Y, Jutzeler CR (2023) Wearable Sensor Technologies to Assess Motor Functions in People With Multiple Sclerosis: Systematic Scoping Review and Perspective. J Med Internet Res 25:e44428
  - Comprehensive overview of studies utilizing wearable sensors to monitor and quantify motor function in people with MS
- Bou Rjeily N, Sanjayan M, Guha Niyogi P, et al (2025) Accelerometry-Assessed Physical Activity and Circadian Rhythm to Detect Clinical Disability Status in Multiple Sclerosis: Cross-Sectional Study. JMIR MHealth UHealth 13:e57599
  - Cross-sectional study utilizing novel analytical approaches to link accelerometry-based activity and circadian patterns with clinical disability in MS
- Goldman MD, Chen S, Motl R, Pearsall R, Oh U, Brenton JN (2023) Progression risk stratification with six-minute walk gait speed trajectory in multiple sclerosis. Front Neurol 14:1259413
  - Longitudinal study utilizing baseline gait speed trajectory to risk stratify people with MS, highlighting the potential of digital tools and sensors as prognostic tools
- Oh J, Capezzuto L, Kriara L, et al (2024) Use of smartphone-based remote assessments of multiple sclerosis in Floodlight Open, a global, prospective, open-access study. Sci Rep 14:122
  - Global open-access initiative highlighting the potential of smartphone-based remote monitoring in large-scale, real-world settings in MS

## Data Availability

No datasets were generated or analysed during the current study.
